# The Theranostics Role of Mast Cells in the Pathophysiology of Rosacea

**DOI:** 10.3389/fmed.2019.00324

**Published:** 2020-01-28

**Authors:** Lian Wang, Yu-Jia Wang, Dan Hao, Xiang Wen, Dan Du, Gu He, Xian Jiang

**Affiliations:** ^1^Department of Dermatology, West China Hospital, Sichuan University, Chengdu, China; ^2^State Key Laboratory of Biotherapy, West China Hospital, Sichuan University and Collaborative Innovation Center of Biotherapy, Chengdu, China

**Keywords:** mast cell, rosacea, pathophysiology, treatment, dermatology

## Abstract

Rosacea is a chronic inflammatory cutaneous disorder that adversely affects patient's health and quality of life due to the complex course and the need for repeated treatment. The exact molecular mechanisms of rosacea are unclear. Mast cells are innate immune cells that can be found in virtually all tissues. Recently, increasing evidence has indicated that mast cells have important effects on the pathogenesis of rosacea. In this review article, we describe recent advances of skin mast cells in the development of rosacea. These studies suggested that mast cells can be an important immune cell that connected innate immunity, nerves, and blood vessels in the development of rosacea. Moreover, we review the inhibition of mast cells for the potential treatment of rosacea.

## Introduction

Rosacea is an inflammatory skin disease that affects between 0.09 and 22.41% of the general population and 5.46% of adults worldwide (Gether, 2018 #100; Parisi, 2018 #202; Parisi, 2018 #202; Parisi, 2018 #202; Parisi, 2018 #202) (1). According to the National Rosacea Society, the primary clinical manifestations of rosacea include transient/persistent erythema and inflammatory papules/pustules with or without telangiectasia (2). Meanwhile, based on clinical features, subtypes include erythematotelangiectatic rosacea (ETR), papulopustular rosacea (PPR), phymatous rosacea (PhR)/rhinophyma, and ocular rosacea. More recently, the term “phenotype,” describing a person's observable characteristics that can be influenced by environmental or genetic factors, was proposed due to the range of manifestations of the multiple subtypes and progression between subtypes (3, 4). The diverse and overlapping clinical manifestations of rosacea have a significant psychological impact on patients, causing self-abasement, depression, anxiety, and social phobia (5). Moreover, rosacea can be a challenging condition due to its complex pathophysiology and progression among subtypes (3, 6).

The exact pathogenesis of rosacea has yet to be fully determined. Major advances have shown that rosacea results primarily from dysregulation of immune responses (innate and acquired immune responses) and/or neurovascular dysfunction with a genetic component (7, 8). Microbes, ultraviolet (UV) radiation, temperature, stress, and a damaged skin barrier can be triggers, stimulating an augmented innate immune response and/or neurovascular dysregulation (6). Cutaneous receptors, including Toll-like receptor (TLR) 2, nucleotide-binding oligomerization domain (NOD)-like receptor, and transient receptor potential vanilloid (TRPV4), are overexpressed in response to these triggers. Activation of these receptors causes the production of innate immune peptides (e.g., LL-37) and diverse cytokines and chemokines, which induce a variety of proinflammatory reactions (9). Moreover, TLR2 can activate the transcription factor nuclear factor kappa B (NF-κB), a mediator that regulates the inflammatory response (10). These events further positively affect the amplification of inflammation and the dysregulation of adaptive immunity and neurovascular changes, which promote physiological responses, including vasodilation, inflammation, fibrosis, and glandular hyperplasia, and the formation of various skin lesions (6).

Mast cells (MCs), which originate from the bone marrow CD34^+^ progenitor cells, reside in virtually all tissues throughout the body, particularly in the skin and mucosal surfaces (11). Therefore, MCs are one of the first cells of the immune system that interact with environmental antigens and allergens and invasive pathogens. Furthermore, studies indicate that MCs acquire the ability of an antigen presentation after activation and are associated with adaptive immunity (12, 13). Indeed, the roles of skin MCs in skin inflammation, which is involved in rosacea, have been highlighted (14). However, to our knowledge, the roles of MCs in rosacea have not yet been thoroughly reviewed. This review discusses the possible role of skin MCs during the process of rosacea, and a perspective on their inhibition for the treatment of rosacea will be summarized.

## MCS Biology and Activation

MCs are hematopoietic cells and represent potential sources for a series of biologically active secreted products, including different kinds of growth factors and cytokines (15). In humans, MCs are divided into two subtypes based on the protease content of secretory granules: MC_T_ with tryptase in human lung MCs and MC_TC_ with both tryptase and chymase in the skin MCs (16). In mice, mucosal MCs (MMCs) are similar to MC_T_, and connective tissue MCs (CTMCs) are similar to MC_TC_ (16). Both MMCs and MC_T_ are inducible and transient, while CTMCs and MC_TC_ are constitutive and long-lasting. Moreover, selective expression of Mas-related G protein-coupled receptor X (MRGPRX) 2 is an important characteristic of MC_TC_ and CTMCs (17, 18). In the current review, skin MCs are of primary focus, therefore both MC_TC_ and CTMCs will be referred to as “MCs” (skin MCs) throughout this text.

The differentiation, survival, and migration of MCs require a stem cell factor (SCF), which can be regulated by keratinocytes (19, 20). Moreover, C-X-C motif chemokine ligand 12 (CXCL12) is a chemoattractant and activator of MCs through its receptor, the C-X-C chemokine receptor (CXCR4). The activation and migration of MCs play important roles in regulating homeostasis (21). The classic pathway of MC activation is immunoglobulin E (IgE)-mediated degranulation. MCs express high-affinity IgE receptor (Fc epsilon RI or FcεRI), which can cross-link IgE with specific antigens and form IgE/FcεRI complexes, resulting in MC activation (22, 23). Other pathways also stimulate MCs (24). For example, stimulation via the IgG and Ig-free light chains (FLC) can induce MC activation as can the combination of pathogen and pattern recognition receptors (PRRs) on MCs. These PRRs include surface TLRs, endosomal TLRs, lectin-like receptor, and the nucleotide-binding oligomerization domain-like receptor family pyrin domain containing 3 (NLRP3) inflammasome (25–28). TLR2-mediated activation not only induces MC degranulation but also stimulates MCs to produce cytokines, chemokines, and eicosanoids. Dectin-1 is a member of the lectin-like receptor family and is mainly found on dendritic cells but can also be expressed by MCs. Dectin-1 can help MCs destroy *Candida albicans* through nitric oxide production in a TLR-2- or dectin-1-dependent pathway. NLRP3 associates with an adaptor protein and apoptosis-associated speck-like protein containing a C-terminal caspase recruitment domain (CARD) to form an inflammasome. After MC exposure to tumor necrosis factor (TNF)-α, the NLRP3 inflammasome induces NF-κB activation (27). In addition, MRGPRX2, complement receptors, neuropeptides (NPs), and neurotransmitter receptors can also participate in the activation of MCs (29, 30). Furthermore, both LL-37 and NPs can activate MCs through MRGPRX2 (31, 32).

Once activated, the MCs can promote the release of different mediators and have a considerable effect on the pathophysiology of diverse inflammatory diseases (24). First, MCs can release histamine, tryptase, and chymase, which are preformed mediators stored in granules (degranulation) that initiate the allergic cascade. Transgranulation, a variant of degranulation, occurs when MCs directly contact other cells and transfer granules into nearby cells by exocytosis (33). Second, effector molecules, including platelet-activating factor and prostaglandins, can be synthesized upon cell stimulation. Furthermore, a series of cytokines, including interleukin (IL)-1, transforming growth factor (TGF)-β, TNF-α, and vascular endothelial growth factor (VEGF), can be released that further activate MCs (34–36). These effectors can participate in numerous inflammatory responses and endothelial dysfunction in the vasculature, altering neural activity and function (37).

## Role of MCS in Rosacea

The role of MCs in a broad group of inflammatory or immune-related conditions, such as asthma, allergic rhinitis urticaria, and rheumatic disease, has been reported previously (38). For the last decade and more, the functional characterization of MCs in rosacea is just emerging. A study by Aroni et al. showed that the number of MCs was significantly higher in lesions than in clinically uninvolved skin in patients with ETR and PPR, and there was a positive correlation between MC density and the duration of rosacea (39). A similar study by Schwab et al. Demonstrated, by quantitative analysis of tryptase staining, that the density of MCs was significantly increased in all subtypes of rosacea, especially PPR. Double immunofluorescence staining revealed that sensory nerves were closely associated with the blood vessels and MCs (40). Another study illustrated that quantitative analysis of tryptase staining revealed a significant increase in the ETR group compared with the control group (41). CXCL12 and its receptor C-X-C chemokine receptor 4 (CXCR4) were significantly higher in the ETR group than in the control group (41). Similarly, a study showed that the mRNA expression of chymase and metalloprotease 9 (MMP9), essential markers of MC presence and activation, was significantly elevated in rosacea skin compared to healthy skin specimens (42). Herein, MCs with their functions and molecular mechanisms characterized in rosacea are discussed in detail.

### MCs Participate in Innate Immune Responses in Rosacea

Studies have demonstrated that MCs can heighten host defense by initiating inflammation associated with innate immune responses. The host defense function of MCs is mainly mediated by rapid degranulation and neutrophil recruitment. Host defense peptides (HDPs), such as cathelicidin LL-37, can cause degranulation via MRGPRX2 (31). HDPs also result in increased expression of TLR4 on MCs, which may strengthen the ability of MCs to detect invading pathogens. In addition, LL-37 can influence MC activity by inducing chemotaxis and the release of proinflammatory cytokines, such as IL-6 and MMP9. Activated MCs can also release MMP9, further promoting the production of LL-37 (42). Muto et al. performed *in vitro* co-culture of normal human epidermal keratinocytes (NHEKs) with supernatant from human MCs (huMCs) previously degranulated by compound 48/80. MMP and kallikrein (KLK) activities were significantly enhanced, and LL-37 mRNA levels were significantly increased. *In vivo*, inflammation was observed in wild-type (WT) mouse skin after the injection of LL-37 (a rosacea mouse model), whereas MC-deficient mice did not show any rosacea-like changes following LL-37 injection. The mRNA expression of MMP9 was significantly lower in MC-deficient mouse skin than in WT mouse skin upon LL-37 injection (42). Similarly, a study by Kim et al. showed that MC number and tryptase and chymase mRNA levels were significantly increased in the skin after the injection of LL-37 compared to the control (43). LL-37 activates MCs, which promote protease production and amplify inflammation, causing an innate immune response that facilitates the development of rosacea.

### MCs Participate in Neurogenetic Inflammation in Rosacea

Dermal MCs are closely associated with neurogenic inflammation and mediate the stress–response network in skin inflammation. Stimulation of MCs by NPs, such as 5-hydroxytryptamine (serotonin) receptor 3A (HTR3A), pituitary adenylate cyclase-activating polypeptide (PACAP), substance P (SP), and vasoactive intestinal peptide (VIP), can cause MC degranulation and the release of histamine, tryptase and other mediators (e.g., TNF-α, CXCL9, CXCL10, and CXCL8), thereby promoting inflammation and resulting in itching, flushing, erythema, and/or burning sensations, which are common symptoms of rosacea. In turn, histamine can lead to the release of NPs at the sensory nerve endings, which creates a bidirectional loop between the MCs and sensory nerves. *In vivo*, tryptase, MMP1a, MMP9, CXCL2, and TNF-α mRNA expression was dramatically increased by NP stimulation in WT but not in MC-deficient mice injected with LL-37 (42). In patients with ETR, the expression levels of HTR3A, PACAP, and VIP were significantly enhanced (40). These results indicated that neurovascular changes and NP release can be translated to rosacea inflammation through the activation of MCs.

TRPV4, one member of the TRPV channels, plays a role in various sensory pathologies (44). Mascarenhas et al. found that TRPV4 was markedly increased in LL-37-injected mice. Immunofluorescence staining also showed a remarkable increase in the co-localization of MC chymase and both TRPV2 and TRPV4. LL-37 directly enhanced TRPV expression in huMCs, and TRPV4 expression is essential for the full degranulation of MCs in rosacea. Moreover, TRPV4 upregulation is dependent on MRGPRX2 in rosacea (45). In addition, the molecular inhibition of TRPV4 for 24 h before irritating cultured MCs with compound 48/80 weakened degranulation, and the knockdown of TRPV4 in huMCs reduced MC degranulation by both compound 48/80 and LL-37 (46). This evidence illustrates that MC activation stimulated by NPs or TRPV4 promotes neurogenetic inflammation associated with rosacea.

### MCs Participate in Vasodilation and Angiogenesis in Rosacea

MCs can produce different proangiogenic molecules, including VEGF, fibroblast growth factor (FGF), histamine, and tryptase. First of all, these molecules directly stimulate the migration and/or proliferation of endothelial cells, thus facilitating vascularization and angiogenesis. Besides, FGFs indirectly degrade the connective tissue matrix to provide space for the formation of neovascular sprouts to promote vascularization, angiogenesis, and telangiectasias (14). In addition, histamine and 5-hydroxytryptamine can bind to the vascular receptors, causing vasodilation and increased vascular permeability (47). Vasodilation leads to erythema, and increased vascular permeability causes flushing. Furthermore, histamine causes increased blood flow and disrupts the endothelial barrier (48). These events suggest that MCs also participate in vasodilation and angiogenesis in rosacea.

### MCs Participate in Fibrosis in Rosacea

Usually, skin fibrosis is clinically present in only advanced stages, especially in PhR (7). Histamine and tryptase, which are released by MCs, enhance fibrosis in rosacea through their chemotactic function on fibroblasts and MMPs (42). MCs have also been shown to promote the proliferation of fibroblasts through VEGF and basic FGF (49). In turn, fibroblasts release SCF, upregulating the expression and synthesis of monocyte chemoattractant protein-1 (MCP-1) in MCs. Moreover, MCP-1 can also act on fibroblasts, facilitating fibrosis (50). MMP activity can increase the fibrillar extracellular matrix, thereby promoting fibrosis (51). These fibrotic effects induce the clinical manifestation of rhinophyma in rosacea.

## Does the Interaction Between MCS and Other Immune Cells Take Part in Rosacea?

Adaptive immunity along with the innate immune system might play a critical role in the pathophysiology of rosacea. Transcriptome analysis on facial biopsies of rosacea patients revealed significant upregulation of macrophages, neutrophils, mast cells, and T helper 1 cell/T helper 17 cell (Th1/Th17)-polarized immune cells (52). Although there are no reports on the interaction between MCs and other immune cells, including macrophages, neutrophils, and T cells in rosacea, many studies have proven that MCs can be activated by other immune cells or can activate neighboring cells through the release of inflammatory mediators (19, 53). Macrophage is an antigen-presenting cell (APC) that is regarded as a significant orchestrator of skin immunity. Neutrophil, typically the first leukocyte to be recruited to an inflammatory site, is capable of eliminating pathogens. Buhl et al. found significant upregulation of macrophage and neutrophils in PPR patients (52). TLR2 and inflammasome complex (IL-1β), accelerating inflammation activation, are expressed on the cellular membrane of macrophages (7). LL-37 and MMP9 are expressed by neutrophils recruited to tissues in response to inflammation (54). On the one hand, macrophages and MCs act as sentinel cells that initiate neutrophil recruitment via inducing increased permeability of local blood vessels and the release of chemokines. One the other hand, MC reconstitution plays an important role in the regulation of neutrophil homeostasis by affecting macrophages (55).

Previous studies revealed that T cells' response takes part in the development of rosacea. CD4^+^ T cells are highly present in the rosacea skin. Th1 cells along with Th17 cells, differentiated from CD4^+^ T cells, are increased in the skin tissues of rosacea (52, 56). The release of interferon-γ (IFN-γ) and IL-17 from the T cells stimulates the production of inflammatory lesions and angiogenesis in rosacea (57). The processing and presentation of antigenic peptides on MCs lead to the expansion of CD4^+^ T cells. In turn, T cells and surface molecules on membrane vesicles secreted can activate MCs, suggesting a bidirectional relationship (58). Based on these evidences in MCs network, we suggest that the possible interaction between MCs and other immune cells especially macrophages, neutrophils, and Th1/Th17 cells may also participate in the pathogenesis of rosacea; although this would require further studies.

## Inhibition of MCS For Rosacea Treatment

### Cromolyn

Cromolyn sodium can inhibit the release of histamine and other inflammatory mediators from sensitized MCs (59). It is widely used in the management of chronic asthma, mastocytosis, seasonal and perennial allergic rhinitis, allergic conjunctivitis, and vernal keratoconjunctivitis (59, 60). *In vivo*, rosacea-like inflammation did not occur in mice pretreated with cromolyn and then challenged with LL-37. The mRNA levels of MMP9 and CXCL2 were significantly reduced in the cromolyn-treated mice compared to those injected with LL-37 alone. MMP activity in the skin was also dramatically weakened by cromolyn pretreatment (42). Furthermore, 10 patients with ETR were chosen to topically apply either 4% cromolyn sodium or placebo on their faces. After 8 weeks, only the cromolyn treatment group showed decreased facial erythema. In addition, MMP activity was markedly decreased in the cromolyn treatment group with mildly decreased KLK activity and LL-37 protein levels (42). These results indicate that cromolyn or other MC stabilizers may be a potential therapy for rosacea, especially ETR, through the inhibition of MC activation.

### Other Drugs Inhibited the Number or Activity of MCs

Brimonidine, a highly selective α2-adrenergic receptor agonist that binds alpha 2 receptors on the vasculature and causes direct vasoconstriction of both small arteries and veins, is approved for the treatment of rosacea with facial erythema by the Food and Drug Administration (61, 62). A study by Kim et al. showed that clinical manifestations of rosacea induced by LL-37 injection in mice were ameliorated after topical application of brimonidine tartrate 0.33% gel, with a noteworthy histological decrease in the number of MCs. Furthermore, the LL-37-induced increases in the mRNA levels of tryptase and chymase were significantly reduced after the use of brimonidine gel (43). Although the relationship between brimonidine and MCs is unclear, these results suggest that the application of brimonidine may decrease the recruitment of MCs in rosacea.

Botulinum toxin (BoNT) A, which is produced by the bacterium *Clostridium botulinum*, can prevent the release of acetylcholine from the presynaptic vesicle and modulate SP and VIP, reducing local inflammation around the nerve endings, deactivating the sodium channel, and exhibiting axonal transport (63). Intradermal BoNT A has been regarded as a novel therapy for ETR and PPR (64, 65). Recently, Choi et al. investigated the direct action of BoNT A on MCs in a rosacea mouse model (66). BoNT A reduced MC degranulation both *in vivo* and *in vitro*. There was significantly less erythema in mice pretreated with intradermal BoNT A than in control mice; both groups were injected with LL-37. Additionally, the intradermal BoNT A pretreatment group displayed a reduction in dermal MC degranulation in comparison with the control (66). This study indicates that BoNT A can reduce rosacea-associated skin inflammation through direct inhibition of MC degranulation.

## Conclusion and Perspective

**Figure 1:**
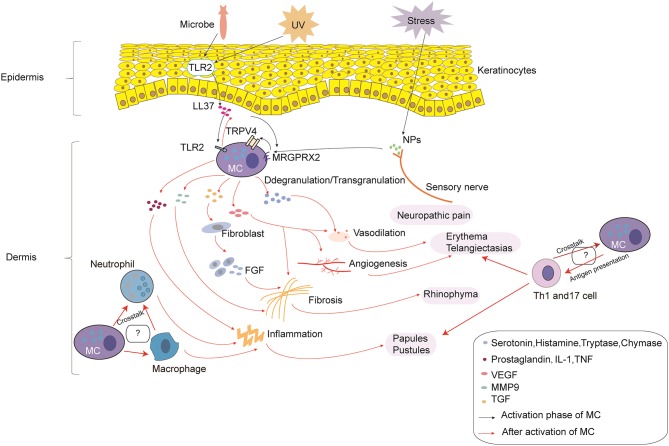
The roles of mast cells (MCs) in the pathophysiology of rosacea. In the process of MC activation, triggers (e.g., microbes and UV) can induce the production of LL-37 by keratinocytes. In addition, stress can induce the production of NPs by sensory nerves. LL-37 promotes the activation of MCs through binding to TLR2 on MCs, which can enhance the production of LL-37. LL-37 and NPs can also promote the activation of MCs through MRGPRX2, which promotes the activation of TRPV4 on MCs. After activation, MC degranulation/transgranulation secretes a series of cytokines and mediators, thereby facilitating vasodilation, inflammation, and angiogenesis, which results in neuropathic pain, erythema, telangiectasias, and the formation of papules and pustules. Moreover, MMP9 produced by MCs directly promotes fibrosis, and cytokines and mediators produced by MCs indirectly promote fibrosis through fibroblasts, which secrete FGF. Furthermore, the cross-talk with macrophages, neutrophils, and Th1/Th17 cells may also take part in the pathophysiology of rosacea, which needs further study.

Rosacea is a complex disorder caused by immune dysfunction and neurovascular dysregulation involving many types of inflammatory cells. In the current review, we describe in detail the roles of MCs in the pathophysiology of rosacea (Figure 1). MCs participate in the pathogenesis of rosacea through innate immune responses, neurogenetic inflammation, angiogenesis, and fibrosis. This review illustrated that MCs can be important immune cells that connect innate immunity, nerves, and blood vessels in the development of rosacea, no matter the subtypes. Although there were no reports on the relationship between MCs and other immune cells, the cellular network indicates that MCs might also play a role in rosacea through interaction with other cells; further studies are needed to focus on this field.

The waxing and waning course of rosacea has led to the necessary development of novel therapies. Cromolyn sodium or other MC stabilizers affect the release of histamine and other inflammatory mediators from MCs to ameliorate erythema in ETR. Drugs including brimonidine and BoNT can also reduce the recruitment of MCs and inhibit MC degranulation to improve inflammation associated with rosacea. We believe that the important role of MCs makes it a potential target for drug therapy in rosacea; more studies are needed to evaluate the efficacy for its inhibition in the treatment of rosacea.

In conclusion, this work makes a clear summary of the relevant literature and a proposal for a mastocyte-focused therapeutic approach. The limitation is that although we concluded that MCs may interact with other cells to facilitate the development of rosacea, discussion of this mechanism in the broader complex relationships is limited in the diseased skin.

## Author Contributions

GH and XJ contributed to determine the content of each section and edited the manuscript. LW, Y-JW, DH, XW, and DD collected the data and wrote each section of the manuscript. All authors read and approved the final manuscript.

### Conflict of Interest

The authors declare that the research was conducted in the absence of any commercial or financial relationships that could be construed as a potential conflict of interest.

## Abbreviations

APC: antigen-presenting cell
BoNT: botulinum toxin
CARD: caspase recruitment domain
CXCL: C-X-C motif chemokine ligand
CXCR: C-X-C chemokine receptor
ETR: erythematotelangiectatic rosacea
FGF: fibroblast growth factor
FLC: Ig-free light chains
FcεRI: Fc epsilon RI
HDPs: host defense peptides
HTR3A: 5-hydroxytryptamine (serotonin) receptor 3A
IFN-γ: interferon-γ
IgE: immunoglobulin E
IL: interleukin
KLK: kallikrein
MC: mast cell
MCP-1: monocyte chemoattractant protein-1
MMP9: metalloprotease 9
MRGPRX: Mas-related G protein-coupled receptor X
NLRP3: the nucleotide-binding oligomerization domain-like receptor (NLR) family pyrin domain containing 3
NF-κB: nuclear factor kappa B
NPs: neuropeptides
PACAP: pituitary adenylate cyclase-activating polypeptide
PhR: phymatous rosacea
PPR: papulopustular rosacea
PRRs: pattern recognition receptors
SCF: stem cell factor
SP: substance P
TGF-β: transforming growth factor
Th1 cell: T helper 1 cell
Th17 cell: T helper 17 cell
TLR: toll-like receptor
TNF-α: tumor necrosis factor-α
TRPV: transient receptor potential vanilloid
UV: ultraviolet
VEGF: vascular endothelial growth factor
VIP: vasoactive intestinal peptide
WT: wild-type.
